# Dr. Ryan and His Pupils

**Published:** 1831

**Authors:** 


### Dr. Ryan and his Pupils.

We have received the report of a meeting of nearly one hundred medical students of the late medical theatre, Brewer Street, held at the above place, on the 30th August, when an address to Dr. Ryan was prepared and signed by 96 students. We regret that neither our plan nor our limits will permit us to give this address and the answer to it, together with the subscribed names:—but as the body of the address is brief, we shall make room for its insertion in this place.

"Sir—It is with great satisfaction and pleasure that we embrace the opportunity presented by the termination of another medical course, to acknowledge the kindness with which you uniformly treated us, as well as the unremitting anxiety you constantly evinced for our professional and general welfare, during the time we had the honor of attending your valuable lectures on medicine, obstetricy, and medical jurisprudence.

By offering you our thanks in this manner, we do not attempt to increase the publicity of your name, already well known as a lecturer, and by works, which are allowed to be unrivalled for accuracy of information and sound opinions, but intend it as a testimonial of our esteem as well as gratitude, which we consider justly due to your abilities and merit." (Signed, &c.)

Dr. Ryan returned a long and eloquent answer, in which the students are highly praised for their assiduity, and attention to the instruction of their teacher—while they are complimented on the progress which they have made in the sciences taught by Dr. Ryan.

---

